# *PLoS Computational Biology:* A New Community Journal

**DOI:** 10.1371/journal.pcbi.0010004

**Published:** 2005-06-24

**Authors:** Philip E Bourne, Steven E Brenner, Michael B Eisen

Welcome to *PLoS Computational Biology,* a community journal from the Public Library of Science dedicated to reporting biological advances achieved through computation. The journal is published in partnership with the International Society for Computational Biology (ISCB). The importance of this partnership is described in the accompanying letter from Michael Gribskov, ISCB president.

What motivates us to start a new journal at this time? Computation, driven in part by the influx of large amounts of data at all biological scales, has become a central feature of research and discovery in the life sciences. This work tends to be published either in methods journals that are not read by experimentalists or in one of the numerous journals reporting novel biology, each of which publishes only small amounts of computational research. Hence, the impact of this research is diluted. *PLoS Computational Biology* provides a home for important biological research driven by computation—a place where computational biologists can find the best work produced by their colleagues, and where the broader biological community can see the myriad ways computation is advancing our understanding of biological systems.

*PLoS Computational Biology* is governed by one overarching principle: scientific quality. This quality is reflected in the editorial board and the editorial staff. The editorial board members are leaders in their respective scientific areas and have agreed to give their valuable time to support a quality journal in their field. Behind the scenes, through a rigorous presubmission process, three quality reviews for each paper, and an acceptance rate below 20%, the editors and staff already knew in the six months since the journal was launched that we were producing a first-rate product. The scientific content is now here for all of you to see and will continue to build in the months and years to come.

Computational biology thrives on open access to biological data.

The inaugural issue of *PLoS Computational Biology* includes a Perspective from Sean Eddy on his belief that we are in the “ante”-disciplinary phase of our science—a time ahead of becoming an organized science and not to be confused with the push toward being “inter”-disciplinary, as many would have us become. Perspectives, and, later, Reviews, will be a regular feature of the journal. This is followed by a series of research articles, each including a synopsis distinct from the scientific abstract, written by the authors for nonexperts. Among these articles, Pagel et al. describe a method for determining pairs of functionally related genes and then use that method to study how these pairs are either gained or lost over evolutionary time; Margalit et al. report on the modeling of transcription factor binding sites and discover 29 such binding sites for C2H2 zinc-finger proteins in *Drosophila melanogaster;* and Ehrenberg et al. model ribosome-mediated transcriptional attenuation and explain the functional differences that result from the structural differences of the leader sequences of the trp and his operons.

The vision we have for *PLoS Computational Biology* as a community journal is first and foremost to support the dissemination of our science in a way that draws attention to the quality, depth, and scope of our best work. That this is an open-access journal is an integral part of this vision. Open access ensures not only that everything we publish is immediately freely available to anyone, anywhere in the world, but also that the contents of this journal can be redistributed and reused in ways that increase their value. Computational biology thrives on open access to DNA sequences, protein structures, and other types of biological data—it is high time that we apply the same principle to our papers and unleash our creativity to develop new and exciting ways to use the scientific literature.

To realize this vision, we need your help. We need you to submit your best work to the journal and encourage colleagues to do so as well. Beyond that, recognize that we are a unique community in that we use information in novel ways. The journal itself is just another information stream that can be processed and analyzed. We have our own ideas for what that means and how it can change the face of publishing, but we want your input too. These are exciting times for biology and publishing. We hope to capitalize on this excitement, so do not hesitate to tell us what we should be doing by sending e-mail to ploscompbiol@plos.org.

